# UK dogs eating raw meat diets have higher risk of *Salmonella* and antimicrobial‐resistant *Escherichia coli* faecal carriage

**DOI:** 10.1111/jsap.13488

**Published:** 2022-02-21

**Authors:** E. F. Groat, N. J. Williams, G. Pinchbeck, B. Warner, A. Simpson, V. M. Schmidt

**Affiliations:** ^1^ Department of Small Animal Clinical Science Institute of Infection Veterinary and Ecological Sciences (IVES), University of Liverpool, Leahurst Campus Liverpool UK; ^2^ Department of Livestock and One Health Institute of Infection Veterinary and Ecological Sciences (IVES), University of Liverpool, Leahurst Campus Liverpool UK

## Abstract

**Objectives:**

To compare detection of *Salmonella* species and antimicrobial‐resistant *Escherichia coli* in the faeces of dogs eating raw meat or non‐raw diets and examine risk factors for their carriage.

**Materials and Methods:**

Canine faecal samples (raw fed n=114; non‐raw fed n=76) were collected from May to July 2015 from across the UK. Enrichment and selective culture and biochemical and PCR assays were used to identify isolates. *Escherichia coli* underwent susceptibility testing to a range of antimicrobials, including third‐generation cephalosporins; PCR assays were used to detect antimicrobial‐resistant genes. Questionnaires were used to collect data on independent variables as risks for antimicrobial‐resistant (resistant to ≥1 tested antimicrobial), multi‐drug‐resistant (resistant to ≥3 antimicrobial classes) and third‐generation cephalosporin resistant *Escherichia coli*.

**Results:**

Antimicrobial‐resistant, multi‐drug‐resistant and third‐generation cephalosporin resistant *Escherichia coli* were significantly more likely to be detected in raw fed (54, 25 and 31%, respectively) compared to non‐raw fed (17, 4 and 4%, respectively) dogs; *Salmonella* species were detected in eight (4%) raw fed dogs only.

**Clinical Significance:**

Raw fed dogs may be a source of *Salmonella* species and *Escherichia coli*, resistant to highest priority critically important antimicrobials, representing a potential animal welfare and public health issue. Owners should be aware of the risks, especially households with members, both human and canine, who are very young, elderly or immunocompromised.

## INTRODUCTION

*Escherichia coli* are the most numerous facultative anaerobic Gram‐negative bacilli in mammalian gastrointestinal tracts and like many commensals, they are potentially pathogenic (Tenaillon *et al*. 2010). Gastrointestinal *E. coli* can also act as reservoirs of antimicrobial‐resistance (AMR) genes for pathogenic variants or other bacteria by horizontal transfer on mobile genetic elements, such as plasmids (Vila *et al*. 2016). AMR and multi‐drug resistance (MDR; resistance to three or more antimicrobial classes) are a growing threat to both human and animal health, increasing reliance on newer generation drugs, including those defined as the highest priority critically important antimicrobials, such as fluoroquinolones and third‐generation cephalosporins (Gould 2009, WHO 2019). Given the close contact between companion animals and their owners, there is an opportunity for the transmission of such bacteria between them (Guardabassi *et al*. 2004).

Extended‐spectrum beta‐lactamase (ESBL)‐producing *E. coli* are of concern as they are resistant to third‐generation cephalosporins (3GCR) and are often MDR (Bush 2018). The reported prevalence of ESBL‐producing *E. coli* in healthy dogs in the UK community is low at 0.5 to 4% (Schmidt *et al*. 2015, Wedley *et al*. 2017); the most commonly encountered β‐lactamases conferring 3GCR found in bacteria isolated commensally and clinically from companion animals are CTX‐M (*bla*_CTX‐M‐15_; *bla*_CTX‐M_ group 1), TEM‐158, SHV‐12 and AmpC (*bla*_CMY‐2_; *bla*_CITM_) enzymes (Ewers *et al*. 2012, Tuerena *et al*. 2016, Wedley *et al*. 2017, Schmidt *et al*. 2018, Zogg *et al*. 2018). AmpC type β‐lactamases are a particular problem as they hydrolyse broad and extended‐spectrum cephalosporins and are not inhibited by β‐lactamase inhibitors such as clavulanic acid (Bush 2018). Transmissible low‐level fluoroquinolone resistance, conferred by *qnr* genes, may also be present in such bacteria (Zogg *et al*. 2018).

*Salmonella* is a significant cause of non‐typhoidal inflammatory gastroenteritis in people. Human illness associated with *Salmonella* is usually attributable to cross‐contamination in the kitchen from raw meat and/or the consumption of inadequately cooked meat (PHE 2013). The prevalence of *Salmonella* in a group of healthy UK dogs from midlands was reported to be low (0.23%; n=436 dogs) (Lowden *et al*. 2015). Similarly, a low *Salmonella* prevalence was reported in a large multi‐state, USA study (n=2422 dogs) in diarrhoeic (1.3%) and non‐diarrhoeic (1.1%) dogs (Reimschuessel *et al*. 2017) and in a UK study (n=160,427 samples) amongst laboratory submitted canine faecal samples (0.82%) (Arsevska *et al*. 2017). On the other hand, a Canadian study (n=138 dogs) reported the detection of faecal *Salmonella* in 23% of dogs where there was a positive association with eating raw meat diets or raw animal products (Leonard *et al*. 2011).

Raw meat diets are increasing in popularity (ACMSF 2018). Previous studies have shown that raw meat fed to dogs can be contaminated with *E. coli* and *Salmonella* species (Nilsson 2015, Baede *et al*. 2017, van Bree *et al*. 2018, Bacci *et al*. 2019, Hellgren *et al*. 2019) and feeding raw meat has been shown to be a risk factor for the detection of canine faecal *Salmonella* species and AMR *E. coli* (Schmidt *et al*. 2015, Wedley *et al*. 2017, Runesvard *et al*. 2020, Viegas *et al*. 2020). The consumption of raw meat by domestic pets may introduce AMR *E. coli* and other pathogenic bacteria into households (Johnson *et al*. 2007, Frost 2017, O'Halloran *et al*. 2020) where they can potentially colonise intestinal tracts and spread between household members by oro‐faecal contamination (Johnson *et al*. 2008). The aim of the present study was to investigate the prevalence of AMR *E. coli* and the enteric pathogen *Salmonella* species in dog faeces prospectively from pets fed raw or non‐raw diets in the UK and to determine risk factors associated with such carriage.

## MATERIALS AND METHODS

### Sample population

Sample size estimates were based on the prevalence of 3GCR *E. coli* in canine faeces. Previous reported data (Wedley *et al*. 2011, Schmidt *et al*. 2015) and the author's unpublished pilot data suggested a prevalence of 40% *versus* <10% for dogs eating raw or non‐raw diets, respectively. To detect a difference between the groups, with 80% power and 95% confidence, it was determined that 32 dogs in each group were required. Based on previous studies, we expected a 75% response rate (Schmidt *et al*. 2015) and, therefore, aimed to recruit between 80 and 150 dogs in each group. Dogs were recruited *via* social media on a first‐response basis between May and July 2015. Participants were sent a sample pack with paperwork (consent form, information sheet and owner questionnaire), sample pot, gloves and biohazard bag. Samples were to be returned *via* prepaid first‐class post the same day as collection. Participation was restricted to one faecal sample per dog and a maximum of two dogs per household for recruitment. An owner questionnaire was administered, which had been used in previous studies (Wedley *et al*. 2011, Schmidt *et al*. 2015). Data were collected on the age, breed, gender, diet and reason for choice, health status (including any perceived health benefit due to diet) and number and type of in‐contact pets in the household. The foods listed on the questionnaire allowed the assignment of each dog to either raw or non‐raw fed groups. A dog was considered to eat raw diet if any component of the diet, fed on a regular basis (minimum once weekly), was not cooked; cooked included commercial kibble, tinned meat, treats and home‐cooked food. Ethical approval for the study was granted from the University's Veterinary Ethics Committee.

### *Escherichia coli* isolation and identification

Faecal samples were tested immediately on receipt in the laboratory and processed as previously reported (Schmidt *et al*. 2015) for *E*. *coli*, including 3GCR isolates. Where present, isolates typical of *E. coli* were randomly selected and sub‐cultured onto nutrient agar: one colony from cefotaxime (CX) (1 mg/L) and/or ceftazidime (CZ) (1 mg/L) eosin methylene blue agar (EMBA) plates, and three colonies from plain EMBA. All isolates were confirmed biochemically as *E. coli* (Schmidt *et al*. 2015).

### *Salmonella* species isolation and identification

Faeces (1 g) were incubated aerobically at 37°C overnight in 5 ml of buffered peptone water for pre‐enrichment and then 100 μl was inoculated into 10 ml of Rappaport‐Vassiliadis broth (RVB) and incubated aerobically at 42°C overnight. RVB cultures were inoculated onto xylose lysine deoxycholate and deoxycholate citrate agars and incubated aerobically at 37°C for 18 to 20 hours. Where present, isolates typical of *Salmonella* species were selected from each plate and sub‐cultured onto chromogenic agar for Salmonella esterase and incubated aerobically at 37°C for 20 to 24 hours. Isolates typical of *Salmonella* species were confirmed with Poly O and Poly H antisera as described by the manufacturer (ProLab, Wirral, UK) using a slide agglutination test.

### *Escherichia coli* antimicrobial susceptibility testing

Antimicrobial susceptibility disc diffusion testing was performed according to the Clinical Laboratory Standards Institute (CLSI 2013) against: tetracycline 30 μg (T30), trimethoprim‐sulfamethoxazole 1.25/23.7 (TS25), ampicillin 10 μg (AP10), co‐amoxyclav 20 μg/10 μg (AUG30), ciprofloxacin 5 μg (CIP5), chloramphenicol 30 μg (C30) and gentamicin 10 μg (GM10). *Escherichia coli* ATCC® 25,922 (LGC Standards, Teddington, UK) was used as a control. Isolates selected from CX or CZ EMBA and isolates with phenotypic resistance to ampicillin or amoxicillin–clavulanate were further screened for 3GCR (cefpodoxime 10 μg) and phenotypic ESBL‐production, according to manufacturer instructions (Extended Spectrum Beta‐Lactamase Set D52C, MAST Group Ltd., Liverpool, UK) using the double‐disc test (M'Zali *et al*. 2000). Interpretation was based on the CLSI guidelines for animal species‐specific zone diameter (millimetre) interpretive standards for veterinary pathogens when available, otherwise human‐derived interpretive standards were used (CLSI 2015). As CLSI standards were not available for interpretation of ciprofloxacin, the European Committee on Antimicrobial Susceptibility Testing zone diameter interpretive standards were used (EUCAST 2015). All media was obtained from Lab M Ltd. (Bury, UK), the antibiotic powder from Sigma–Aldrich Company Ltd. (Gillingham, UK) and all antimicrobial discs from MAST Group Ltd. (Liverpool, UK).

### DNA extraction

To extract DNA (*E. coli*), three colonies from each overnight culture were homogenised in 500 μl of sterile distilled water and heated at 100°C for 10 minutes before being centrifuged at 13,000 rpm for 2 minutes to retain the supernatant. All DNA extractions were stored at −80°C before use.

### Genotypic characterisation of *E. coli*

A PCR assay was used to detect the presence of the *uid*A gene to confirm the identification of *E. coli* (McDaniels *et al*. 1996). *Escherichia coli* isolates with phenotypic ESBL‐production ±resistance to amoxicillin–clavulanate were tested for the presence of *bla*_CTX‐M_ (Batchelor *et al*. 2005), *bla*_SHV_, *bla*_TEM_ and *bla*_OXA_ (Dallenne *et al*. 2010) and *bla*_AmpC_ gene (Perez‐Perez & Hanson 2002) and the presence of *qnrA*, *qnrB* or *qnrS* genes (Robicsek *et al*. 2006), by PCR assay. Isolates positive for *bla*_CTX‐M_ were tested by additional PCR assay to determine if they belonged to CTX‐M group 1, 2 and 9 genes (Batchelor *et al*. 2005, Hopkins *et al*. 2006). All PCR assays were performed with 5 μl of bacterial DNA, 5 pmol of each primer, 4 μl of 5xFIREPol® Master Mix (12.5 mM MgCl_2_), 0.5 μl of FIREPol® DNA Polymerase 5 U/μl (Solis‐Biodyne, Tartu, Estonia) and water, made up to a total reaction volume of 25 μl. Positive control strains were included, and molecular grade water (Sigma‐Aldrich Company Ltd.) was used as the negative control. PCR products were analysed by agarose gel (1.5%) electrophoresis and the DNA fragments were visualised under UV light after peqGREEN (peqlab, Fareham, UK) staining.

### Statistical analysis

Outcome data for AMR were collapsed to the sample level such that a sample with at least one isolate that was resistant to a tested antimicrobial was classed as resistant for analysis. The prevalence of resistance was classified as AMR (resistance to at least one tested antimicrobial), MDR (resistance to three or more antimicrobial classes) and 3GCR. The percentage of samples with resistant *E. coli* was calculated with 95% confidence intervals (CI) for each group (raw fed and non‐raw fed) and compared using chi‐squared tests or Fisher's exact test if n ≤5. The detection of *Salmonella* species from raw *versus* non‐raw fed dogs was compared using Fisher's exact test (MedCalc© 2021). Independent variables were created from the owner questionnaires. Except for the age of the dog, all variables were categorical. The three binary considered outcomes were AMR, MDR and 3GCR, and univariable logistic regression analysis examined the association between independent variables and outcomes. All variables P‐value <0.25 were assessed in multi‐level, multi‐variable models with household included as a second‐level random intercept term to account for the clustering effect of sampling multiple dogs from common homes. Final multi‐variable multi‐level models were constructed by manual backwards stepwise procedures where variables with a P‐value <0.05 (calculated using the Wald test) were retained. Data were analysed using the MLwiN statistical software package (version 2.36, Centre for Multilevel Modelling, University of Bristol, UK) (Rabash *et al*. 2016).

## RESULTS

### Sample population and questionnaire data regarding owner's diet choice and reported health benefit

In total, 190 faecal samples were collected from dogs from n=140 households from across the UK: 114 raw fed and 76 non‐raw fed. From the owner‐compiled questionnaires, the most common reason for choosing a raw diet was adverts (28%), friends (26%) and dermatitis (11%). Reasons for choosing a non‐raw meat diet were friends (29%), veterinary advice (24%) and breeder advice (22%) (Table 1). Owners feeding raw diets perceived improvements in their dog's stool consistency (83%), oral hygiene and/or breath (65%) and demeanour/behaviour (39%), compared to a non‐raw diet. Owners feeding a non‐raw diet perceived improvement in their dog's coat quality (51%), stool consistency (41%) and oral hygiene and/or breath (16%) (Table 2). Most owners feeding raw, reported feeding chicken (86%), red meat (86%) and tripe (83.3%), but also bones (19.3%), kitchen leftovers (14.9%) or cured meats, for example, pig's ears (12.3%); most dogs were fed a mixture of meats with the majority (63%) being fed raw tripe, chicken and red meats. Only two dogs were fed a single type of raw food, one being fed raw tripe and another raw chicken.

**Table 1:** Questionnaire data from owners of non‐raw fed (n=76) and raw fed (n=114) dogs: owner‐stated reasons for diet choice

Current diet	Number and percentage of owners			
	Non‐raw (n)	%	Raw (n)	%
Allergies	5	6.6	4	3.5
Dermatitis	6	7.9	13	11.4
Loose stools/colitis	2	2.6	3	2.6
Anal sacs	0	0	0	0
Weight gain	3	3.9	0	0
Renal problems	1	1.3	0	0
Pancreatitis	1	1.3	0	0
Sore ears	0	0	1	0.9
Hot spots	0	0	1	0.9
Dental	0	0	0	0
Joint problems	2	2.6	0	0
Seizures	0	0	1	0.9
Breeder advice	17	22.4	11	9.6
Vet advice	18	23.7	6	5.3
Advertisements	2	2.6	32	28.1
Social media	2	2.6	4	3.5
Friend's advice	22	28.9	30	26.3

**Table 2:** Questionnaire data from owners of non‐raw fed (n=76) and raw fed (n=114) dogs: owner's perceived benefits of the fed diet

Current diet	Number and percentage of owners			
	Non‐raw (n)	%	Raw (n)	%
Coat quality	39	51.3	40	35.1
Stool consistency	31	40.8	95	83.3
Demeanour/behaviour	9	11.8	44	38.6
Oral hygiene and/or breath	12	15.8	74	64.9

### Prevalence of *Salmonella* species and resistant *E. coli*

*Salmonella* species was detected from 4% of all dogs (8/190), all being raw fed. AMR *E. coli* (resistant to at least one tested antimicrobial) were detected from 40% (75/190) and MDR *E. coli* from 16% (31/190) of dogs. Resistance to antimicrobials was significant in raw fed, compared to non‐raw fed dogs, including MDR (Table 3). Resistance to four antibiotic classes was detected in a small number of dogs (n=4), all of which were raw fed dogs. ESBL‐producing *E. coli* (n=52) were detected from the faeces of 35 raw fed and three non‐raw fed dogs.

**Table 3:** The percentage of dogs (n=190) with at least one faecal *Escherichia coli* isolate resistant to the tested antimicrobials or *Salmonella* species detected

Antibiotic‐resistance phenotype	Raw (n=114)	%	95% CI	Non‐raw (n=76)	%	95% CI	P‐value
TS	32	28.1	19.8 to 36.3	4	5.3	0 to 10.3	<0.001*
Amp	43	37.7	28.8 to 46.6	9	11.8	4.6 to 19.1	<0.001**
AC	4	3.5	0 to 6.9	3	3.9	0 to 8.3	0.45*
GM	2	1.8	0 to 4.2	1	1.3	0 to 3.9	1.0*
Tet	53	46.5	47.3 to 55.6	10	13.2	5.6 to 20.8	<0.001**
Chlor	8	7.0	2.3 to 11.7	2	2.6	0 to 6.2	0.32*
Cip	0	0	0	0	0	0	NA
AMR	62	54.4	45.2 to 63.5	13	17.1	8.6 to 25.6	0.001**
3GCR	35	30.7	22.2 to 39.2	3	3.95	0 to 8.3	0.001*
MDR	28	24.6	16.7 to 32.4	3	3.95	0 to 8.3	0.001*
*Salmonella*	8	7	3.6 to 13.2	0	0	0	0.025*

95% CI Confidence interval, TS Trimethoprim‐sulfamethoxazole resistance, Amp Ampicillin resistance, AC Amoxicillin–clavulanate resistance, GM Gentamicin resistance, Tet, Tetracycline resistance, Chlor Chloramphenicol resistance, NA Not applicable, AMR Antimicrobial resistance (resistance to ≥1 tested antimicrobial), 3GCR Third‐generation cephalosporin resistance; MDR Multi‐drug resistance (resistance to ≥3 antimicrobial classes)

P‐values are from *Fisher's exact or

** Chi‐squared P‐value; significant at P‐value <0.05

### Resistance genes amongst phenotypic ESBL‐producing‐*E. coli*

The most common beta‐lactamase‐encoding genes were *bla*_CTX‐M_ and *bla*_TEM_; however, no *bla*_SHV_ genes were detected. Of the *bla*_CTX‐M_ genes, group 1 genes were the most prevalent. Over a third of the tested isolates carried *bla*_AmpC_ genes and low‐level plasmid‐mediated fluoroquinolone‐resistance genes (Table 4).

**Table 4:** Antimicrobial resistance genes detected in canine faecal ESBL‐*E. coli* (n=52)

Genes detected	Number (%) of isolates
*bla*_TEM_	21 (40)
*bla*_SHV_	0
*bla*_OXA_	11 (21)
*bla*_CTX‐M_	25 (48)
*bla*_CTX‐M_ uncharacterised	5 (10)
*bla*_CTX‐M_ group 1	19 (37)
*bla*_CTX‐M_ group 2	0
*bla*_CTX‐M_ group 9	1 (2)
*qnr*	20 (38)
*qnr*A	10 (19)
*qnr*B	0
*qnr*S	10 (19)
*bla*_AmpC_	17 (33)
*bla*_CITM_	13 (25)
*bla*_ACC_	2 (4)
*bla*_DHA_	2 (4)

### Multi‐level multi‐variable models

Final multi‐variable results (Table 5) showed that compared to non‐raw fed dogs, faecal *E. coli* from raw fed dogs were more likely AMR, MDR and 3GCR. Dogs receiving antimicrobials in the previous 3 months had increased odds of carriage of MDR *E. coli*.

**Table 5:** Multi‐level multi‐variable logistic regression model results for the outcomes “MDR”, “3GCR” and “AMR” in faecal samples from n=190 [raw meat fed (n=114) and non‐raw meat fed (n=76)] dogs

Final multi‐variable results	MDR			3GCR			AMR		
	OR	95% CI	P‐value	OR	95% CI	P‐value	OR	95% CI	P‐value
Feeds non‐raw	Ref	–	–	Ref	–	–	Ref	–	–
Feeds raw	**7.46**	2.13 to 11.39	0.002	**14.57**	3.03 to 70.04	0.001	**6.21**	2.99 to 12.92	<0.001
AB not received in last 3 months	Ref	–	–	–	–	–	–	–	–
Received AB in last 3 months	**3.33**	0.98 to 11.39	0.05	–	–	–	–	–	–
Age (years)	–	–	–	**0.58**	0.41 to 0.83	0.003	–	–	–
Age (years) squared	–	–	–	**1.03**	1.01 to 1.06	0.009	–	–	–
Estimate of household variance (SE)	0.28 (0.63)			1.24 (0.82)			0.22 (0.42)		

Household (n=140) was included as a second‐level random intercept term to account for the clustering effect of sampling multiple dogs from common homes. P‐values are from the Wald chi‐square test; significant at P‐value < 0.05 (odd ratio in bold text)

MDR Multi‐drug resistant (resistant to ≥3 antimicrobial classes), 3GCR Third‐generation cephalosporin resistant, AMR Antimicrobial resistant (resistant to at least one tested antimicrobial), OR Odds ratio, CI Confidence interval, Ref Reference category, AB Antibiotic, SE Standard error

**FIG 1:**
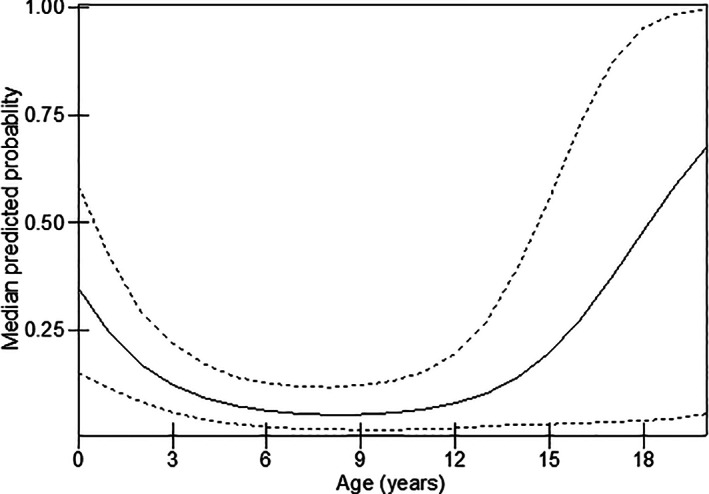
Predicted probabilities of 3GCR and dog age (in years) (predicted probabilities are from the multi‐level, multi‐variable model in Table 5). The solid line is the predicted probability, and the dotted lines are the 95% confidence intervals

Age of dog in years was associated with 3GCR‐resistant *E. coli* carriage, but this was not a linear relationship and was best explained by a quadratic polynomial. This indicated higher predicted probabilities in younger dogs followed by a decrease until approximately 12 years of age when the odds increased again (although confidence intervals here were wider) (Fig 1).

## DISCUSSION

The present study investigated whether feeding raw or non‐raw meat diets to pet dogs affected the detection of *Salmonella* species and/or AMR faecal *E. coli* as carriage of such bacteria may be a health risk to the pet, their owners, or in‐contact pets or people. A significantly higher prevalence of AMR, MDR and 3GCR/ESBL‐producing *E. coli* and *Salmonella* species were detected in dogs fed raw compared to non‐raw meat diets; *Salmonella* species were only found in the faeces of dogs eating raw meat diets.

The present study identified an overall prevalence of MDR *E. coli* of 16% out of 190 dogs (95% CI: 11.1 to 21.6), which was like the prevalence of MDR *E. coli* reported previously in a large cross‐sectional cohort of healthy UK‐based dogs, in which being fed raw poultry was reported as a risk factor for MDR *E. coli* (Wedley *et al*. 2017). Another UK study reported a higher prevalence (30%) of MDR faecal *E. coli* in healthy dogs (Schmidt *et al*. 2015) and again, raw meat diets were significant. In the present study, MDR *E. coli* was seven times more likely to be detected from dogs fed raw compared to non‐raw fed dogs.

Our study agreed with other reports from the UK (Wedley *et al*. 2011, Schmidt *et al*. 2015, Wedley *et al*. 2017, Wareham 2019) of common tetracycline and ampicillin resistance amongst canine faecal *E. coli*. In the present study, such phenotypes were significantly increased in raw fed dogs, with ampicillin resistance detected from 37.7% of raw and only 11.8% of non‐raw fed dogs and tetracycline resistance from 46.5% of raw and only 13.2% of non‐raw fed dogs. Wareham (2019) also reported raw meat fed puppies (n=223) had a greater risk of tetracycline‐resistant faecal *E. coli*. Tetracyclines and beta‐lactams (mainly penicillins) are first‐line antimicrobials and comprise most of the total tonnes of antibiotics sold for food‐producing animals in the UK (Eckford *et al*. 2013, UK‐VARSS 2020 2019).

Interestingly, ciprofloxacin resistance was not detected in any *E. coli* in the current study, despite finding genes that provide low‐level quinolone resistance (*qnrA* and *S*). The negative finding may be because fluoroquinolones are not as widely used in livestock, accounting for only 0.6% of total antibiotics sales in the UK over a similar time (Eckford *et al*. 2013) and even less so (0.4%), in 2019 (UK‐VARSS 2020 2019). A more recent study, however, reported that puppies fed raw meat diet had a greater risk of ciprofloxacin resistance (Wareham 2019). The difference in findings between the studies may be explained by the recruitment period (2015 *versus* 2017 to 2018) and/or the methodologies (disc diffusion *versus* minimum inhibitory concentration) used and/or the age group (pups *versus* adults) investigated.

Dogs eating raw meat diets had a higher prevalence for carriage of 3GCR/ESBL‐producing faecal *E. coli* (c.30.0%) compared to other UK‐based studies (Wedley *et al*. 2011, Schmidt *et al*. 2015); however, in previous studies, the prevalence was not differentiated based upon diet. Furthermore, the prevalence in the current study was even higher than that reported in hospitalised dogs in the UK, with a 14% (95% CI: 5.3 to 35.0) ESBL‐*E. coli* prevalence (Tuerena *et al*. 2016). Phenotypic 3GCR/ESBL‐producing *E. coli* isolates often carried *bla*_CTX‐M_, mainly *bla*_CTX‐M_ group 1, in agreement with other UK studies (Schmidt *et al*. 2015, Tuerena *et al*. 2016, Wedley *et al*. 2017). ESBL‐producing *E*. *coli* are often associated with several infections in dogs (Zogg *et al*. 2018), which may decrease the success of treatment and increase the potential for morbidity and mortality (De Krak*er et a*l. 2011); one recent USA study has confirmed similar strains in raw meat samples and companion animal clinical isolates (Jones *et al*. 2019).

Multi‐level modelling results confirmed that after adjusting for clustering and other factors, a raw fed diet was a significant risk factor for AMR, MDR and 3GCR *E. coli*. Eating raw chicken was also identified as a risk factor for the detection of ESBL‐producing *E. coli* in a large UK cross‐sectional study in healthy dogs (n=560) (Wedley *et al*. 2017). In addition, such bacteria have been detected in UK supermarket raw meats (Randall *et al*. 2017) and other studies have reported detection of ESBL‐producing *E. coli* in raw pet food products from the Netherlands, Italy and Sweden (Nilsson 2015, Baede *et al*. 2017, van Bree *et al*. 2018, Bacci *et al*. 2019). Although suppliers of raw meat pet food may advertise “human‐grade raw meat” (Ahmet 2017), it is still raw, and humans should not consume it without proper cooking to kill potentially harmful microorganisms (FSA 2019). Moreover, the packaging of these products generally lacks information on hygienic handling and preparation and in some cases may be damaged, resulting in contamination of hands and/or preparation and storage facilities (Bojanic *et al*. 2017, van Bree *et al*. 2018). Following a serious outbreak of shiga‐toxin‐producing *E. coli* O157, linked to feeding raw meat (PHE 2018), Public Health England has developed online raw meat handling guidelines for pet owners (PHE 2019).

The present study had several limitations. The answers from owner's questionnaires must be analysed with caution, as interpretation of questions and keywords, for example, diarrhoea may be different from veterinarians' definitions. Furthermore, recall may not be accurate when asking questions in terms of previous exposures such as prior antimicrobial treatments. As recruitment may have been biassed by participant's willingness to be involved, the study population may not be representative of all raw fed or non‐raw fed UK dogs. There is no standardised raw meat diet for dogs in the UK. The wide variation in raw diets with respect to composition, source, preparation, packaging and storage may alter the risk for each dog.

In conclusion, dogs fed a raw meat diet had an overall greater prevalence of faecal *Salmonellae* and AMR, MDR and 3GCR/ESBL‐producing *E. coli* than dogs fed non‐raw meat diets. Human exposure to pathogenic and/or AMR bacteria may occur through handling and preparation of raw meat. Strategies should be implemented to increase pet owners' awareness of the risks involved with feeding raw meat to their dogs and hence reduce any potential risk to themselves, their family and their pets. Hand hygiene should be strongly recommended after handling raw meat along with sanitisation of all in‐contact items as per normal handling of raw meat in a domestic kitchen. The current study provides evidence that can be used to educate owners on the risks of feeding raw meat diets to dogs.

### Funding

Ellyn Groat was funded by PetSaver's 40th Anniversary Veterinary Student Grant and received a stipend for her summer project from the Institute of Veterinary Science, University of Liverpool.

### Conflict of interest

None of the authors of this article has a financial or personal relationship with other people or organisations that could inappropriately influence or bias the content of the paper.
